# Supplementary material for: Distinct core promoter codes drive transcription initiation at key developmental transitions in a marine chordate
Source: BMC Genomics. 2018 Feb 26;19:164. doi: 10.1186/s12864-018-4504-5 (PMC6389100; doi:10.1186/s12864-018-4504-5)
Supplement: Supplementary file 1 — Supplementary Material. Supplemental Results, Supplemental Methods, Table S1, Table S2, Figures S1 - S12 and Supplemental References. (PDF 13 464 kb) [file 12864_2018_4504_MOESM1_ESM.pdf]

Distinct core promoter codes drive transcription initiation at key developmental transitions in a marine chordate

Gemma B. Danks, Pavla Navratilova, Boris Lenhard, Eric M. Thompson

Supplementary Material

**Supplemental Results**	**2**
**Supplemental Methods**	**2**
**Table S1.**	**3**
**Table S2.**	**3**
**Figure S1.**	**4**
**Figure S2.**	**5**
**Figure S3.**	**6**
**Figure S4.**	**7**
**Figure S5.**	**8**
**Figure S6.**	**9**
**Figure S7.**	**10**
**Figure S8.**	**11**
**Figure S9.**	**12**
**Figure S10.**	**13**
**Figure S11.**	**14**
**Figure S12.**	**15**
**Supplemental References**	**15**

Supplemental Results

Increased DNA methylation at TSSs of TATA-dependent promoters in zebrafish

In order to determine if TATA-associated DNA-methylation is a feature conserved between *O. dioica* and vertebrates we analysed previously published data from early zebrafish embryos consisting of high-resolution mapping of zygotic TSSs (CAGE) as well as H3K4me3 and H2A.Z occupancy [1,2] and zygotic DNA methylome profiling (meDIP-seq) [3]. An association between TATA-dependent promoters and an increase in DNA-methylation was present in the proximal region around the TSS (a region comparable to that seen in *O. dioica*) in early embryogenesis (Figure S12A), which increased in later development (Figure S12B). More distal DNA methylation levels converged to similarly high levels (with a slight increase at TATA-dependent promoters in the downstream region), whereas in *O. dioica* they remained substantially higher in TATA-dependent promoters (Figure S11B). This may be a consequence of a highly compacted genome in *O. dioica* (19x smaller than the zebrafish genome) since transcription-associated DNA methylation in other promoter types, with H3K4me3 at the TSS, may not reach a comparable level due to the reduced length of DNA sequence resulting from short introns (mean intron length is 47 bp in *O. dioica*). As in *O. dioica*, and in line with a lower level of DNA methylation, H3K4me3 was depleted at the TSS in TATA-dependent promoters in zebrafish compared to sharp TATA-less promoters (Figure S12C). We also found that levels of H2A.Z, which strongly anti-correlate with CpG DNA methylation [4], were also lower in TATA-dependent promoter TSSs (Figure S12D). Our results suggest that an association between TATA-dependent TSS selection and increased DNA methylation is conserved between vertebrates and invertebrates, although the location in relation to the TSS differs.

Supplemental Methods

Zebrafish data analysis

We used publicly available CAGE data [1,2] from two stages of zebrafish embryogenesis: dome/30% epiboly stage (4.33-4.66 hpf) and prim 6 (24 hpf). We used the R package "CAGEr" [5] to cluster CTSSes into CAGE tag clusters, excluding those with < 1 tpm and singletons < 5 tpm, using a maximum distance of 20 bp between CTSSes within a cluster. We calculated the interquartile range ($q_{0.1}$-$q_{0.9}$) of promoter widths and classed any promoter < 10 bp as sharp following (Nepal et al. 2013). We used the upper quantile of interquartile promoter widths to define broad promoters. We then excluded maternal TSSs according to definitions based on CAGE data across 12 stages of embryogenesis [1,2]. We filtered TSSs further to only include those supported by >= 1 tpm, those associated with protein coding genes and those without any other TSSs in a +/- 2 kb flanking region. We assigned promoters as TATA-dependent if they contained TATAA (similar results were obtained using the consensus TATA motif TATAWAWR). We used published meDIP-seq data (GSE52703) [3] from zebrafish embryos at 4.5 hpf and 24 hpf and published H3K4me3 and H2A.Z ChIP-seq subtracted coverage data [1,2] from the dome/30% epiboly stage. We plotted the mean read count at each bp position in a +/-2kb window around TSSs for different promoter types.

Sample	Number of reads	% uniquely mapping (million reads)	% multiple mapping	% failed to align	Number of tag clusters	% assigned to annotated genes
Female D6	3,886,540	60.78 (2.4)	12.00	27.22	12945	76.27
Male D6	9,238,422	63.58 (5.9)	14.54	21.88	13562	77.74
Oocyte	4,276,690	58.87 (2.5)	10.68	30.46	6455	78.87
Tailbud	5,450,443	58.52 (4.5)	12.83	28.64	11433	70.78
Tadpole	8,244,476	63.76 (5.3)	11.33	24.91	13177	71.64
D2	7,625,608	53.95 (2.9)	14.56	31.48	12647	71.84

Table S1.
CAGE read mapping at different developmental stages.

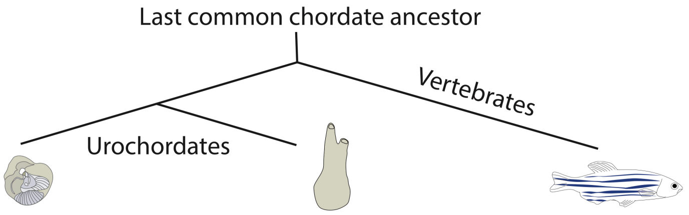

Promoter type	*O. dioica*	*C. intestinalis*	*D. rerio*	Refs
**TCTAGA**	Male-specific	Absent	Absent	
**Maternal**	Broad, no TATA-like elements, ordered nucleosomes, E2F1 binding, *trans*-spliced	*Trans*-spliced	Sharp/multiple sharp with TATA-like elements, disordered nucleosomes	1, 6
**Zygotic (Developmental)**	Broad, TFAP4-like binding sites, not *trans*-spliced	Not *trans*-spliced	Broad, ordered nucleosomes	1, 6
**TATA**	Sharp, Adult (tissue)-specific, increased gene body DNA methylation	Sharp, tissue-specific	Sharp, tissue-specific, increased TSS DNA methylation	1, 2, 7
**RP**	Broad, CpG-rich, TCT initiator absent, no TATA, *trans*-spliced	Sharp, TCT initiator, no TATA, not *trans*-spliced	Sharp, TCT initiatior	2, 6, 7
**Ubiquitous**	Broad with GAAA signal at expected +1 nucleosome position. *Trans*-spliced. Sharp subset with ACCATAA element.	Broad, no CpG enrichment	Broad, CpG-rich	1, 2, 7

RP = Ribosomal Protein gene promoter

Table S2.
Comparison of core promoter features of different promoter types in different chordate species

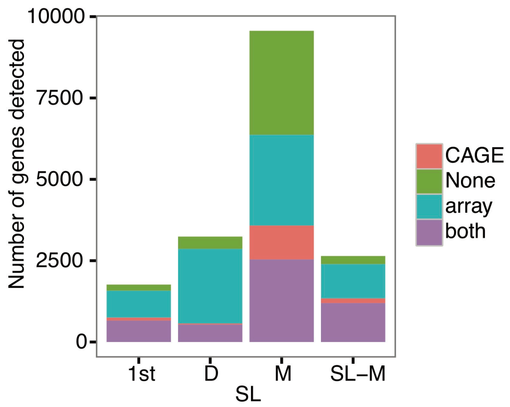

Figure S1.

The number of annotated genes detected by both CAGE and tiling arrays, CAGE only, tiling array only and those not detected by either method. CAGE libraries were depleted of *trans*-spliced transcripts and CAGE is unable to profile downstream operon genes unless they are transcribed from internal promoters. M = monocistronic (not *trans*-spliced); SL-M = *trans*-spliced monocistronic; 1st = first gene in an operon; D = downstream operon gene.

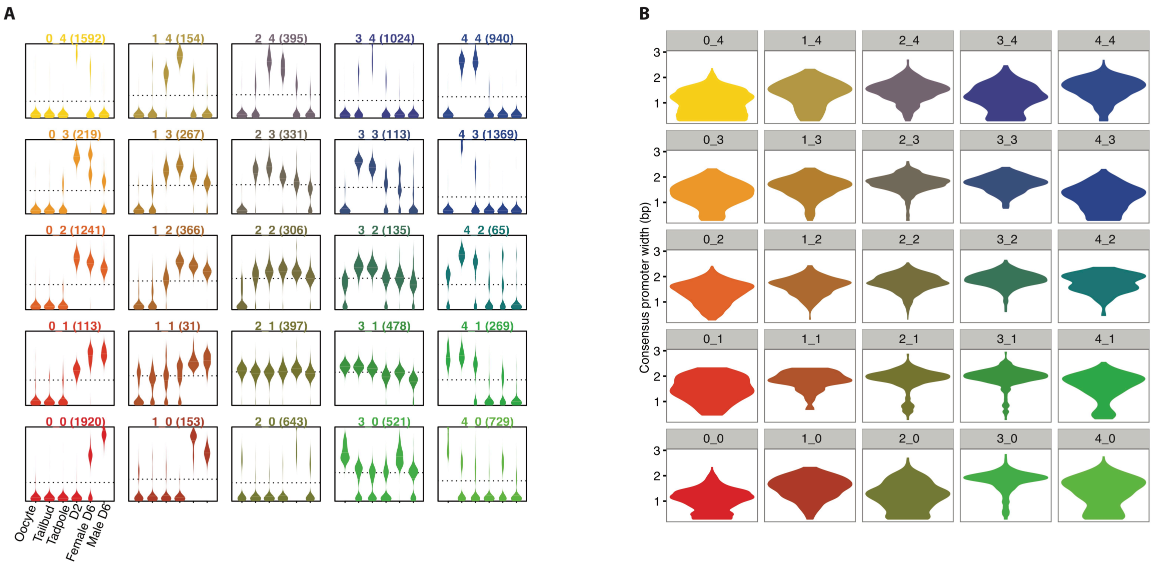

Figure S2.

Expression profiles obtained from self-organising map clustering of consensus promoter regions. **(A)** Each beanplot shows the distribution of relative expression of genes from promoters (number above each plot) within each cluster at each developmental stage (x-axis) indicated in the bottom left plot. **(B)** Beanplots show the distribution of promoter widths within each expression cluster in (**A**).

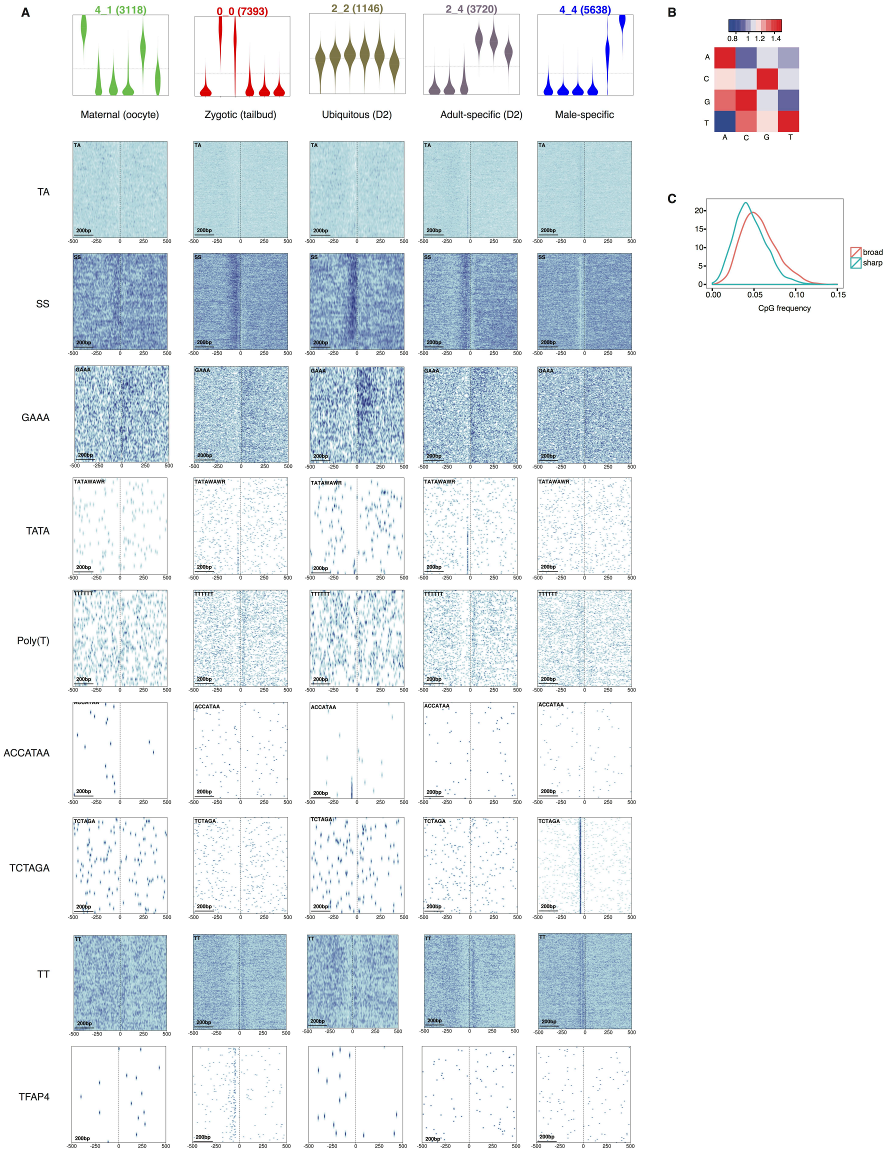

Figure S3.

Dinucleotide content and sequence motifs of *O. dioica* promoters. (A) Each heatmap shows the density of the dinucleotide or motif, as indicated (left), at each position (x-axis) in the -500 to +500 bp region centred on the dominant TSS, for promoter sequences (rows) ordered by promoter width (top to bottom = broad to sharp). Darker blue indicates higher enrichment. Plots are shown for main expression clusters defined as indicated (top; expression profile for each also shown in Figure 1C). Promoter sequences and TSS positions were taken from representative/dominant stages as indicated in parentheses (top). (B) Heatmap of observed/expected ratios for all dinucleotide frequencies in the genome of *O. dioica* where red represents greater than expected and blue less than expected. The genome is most depleted of TA dinucleotides; it is most enriched for TT, AA, CG and GC. (C) Broad promoters have a higher CpG content than sharp promoters. Density plot shows the distribution of CpG frequencies (x-axis) in a 200 bp window centered on the dominant CTSS across all broad (pink) and sharp (blue) promoters.

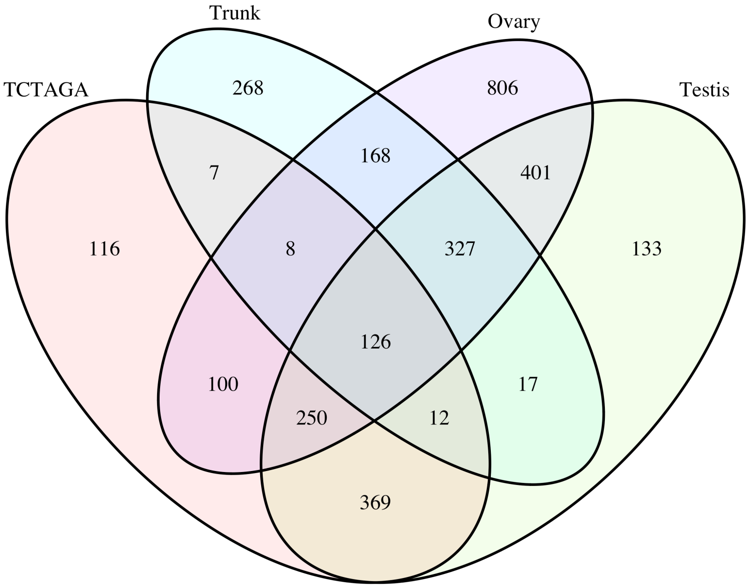

Figure S4.

The majority of testis-specific genes are associated with a TCTAGA promoter element. Venn diagram shows the overlap of genes expressed (as detected by microarray) in dissected ovaries, testis and day 6 trunks (male and female animals after dissection of gonads) with genes that have a TCTAGA promoter element. Only genes with promoters detected by CAGE are included in the analysis.

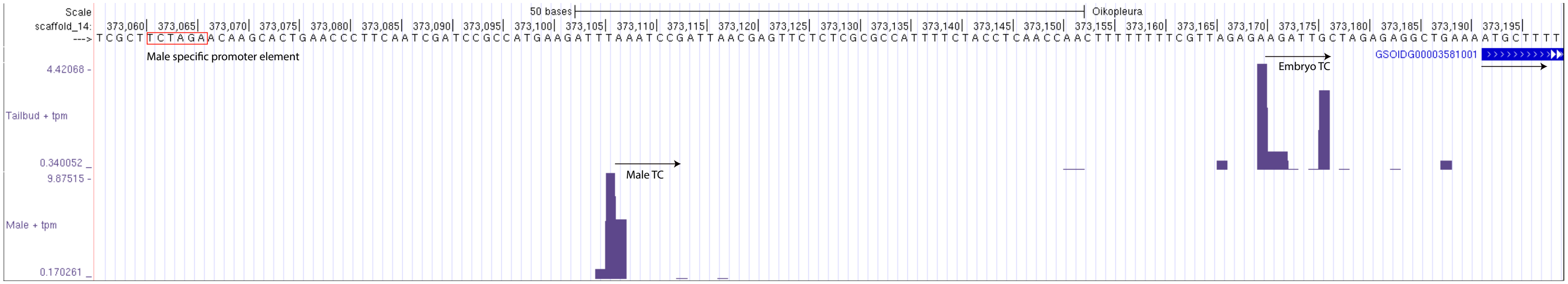

Figure S5.

Shifting promoters. Genome browser screen shot shows CAGE tracks from day 6 male (lower track) and tailbud (embryo; upper track) samples at a shifting promoter. Height of bars gives tag counts per million reads (tpm) at each bp corresponding to a TSS.

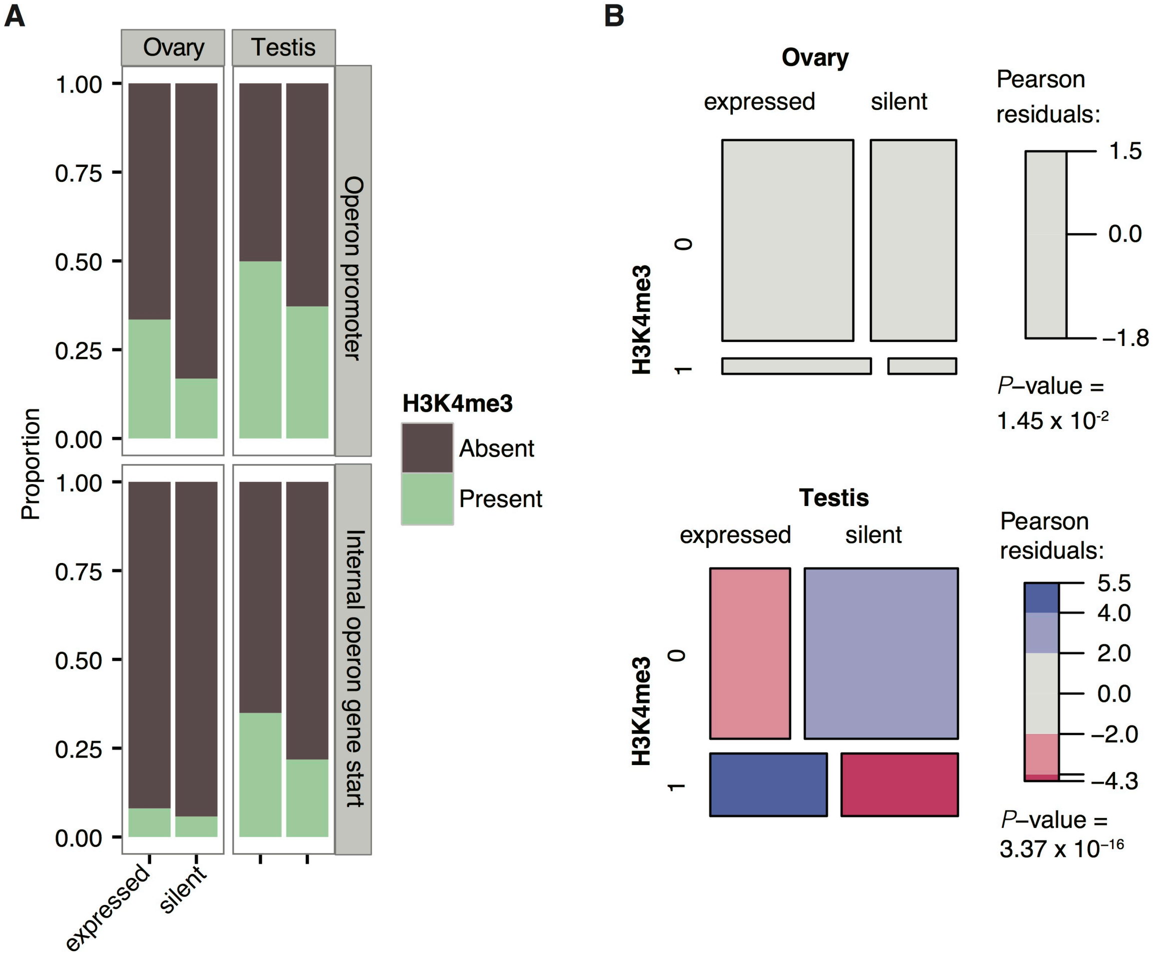

Figure S6.

Two modes of transcription for operon genes in the *O. dioica* ovary and testis. (A) Promoter regions of expressed operons are enriched for the active promoter mark H3K4me3 in both the ovary and the testis (upper panels). The start sites of downstream operon genes are also enriched for H3K4me3 in the testis only (lower panels), indicating cryptic male-specific internal promoters within operons. Each panel shows the proportion of expressed and silent genes/operons with a promoter region that overlaps an H3K4me3 ChIP-enriched region. **(B)** Mosaic plots visualizing the results of Pearson chi-squared tests for the association of H3K4me3 in internal operon gene promoter regions in the ovary (not significant) and testis (highly significant).

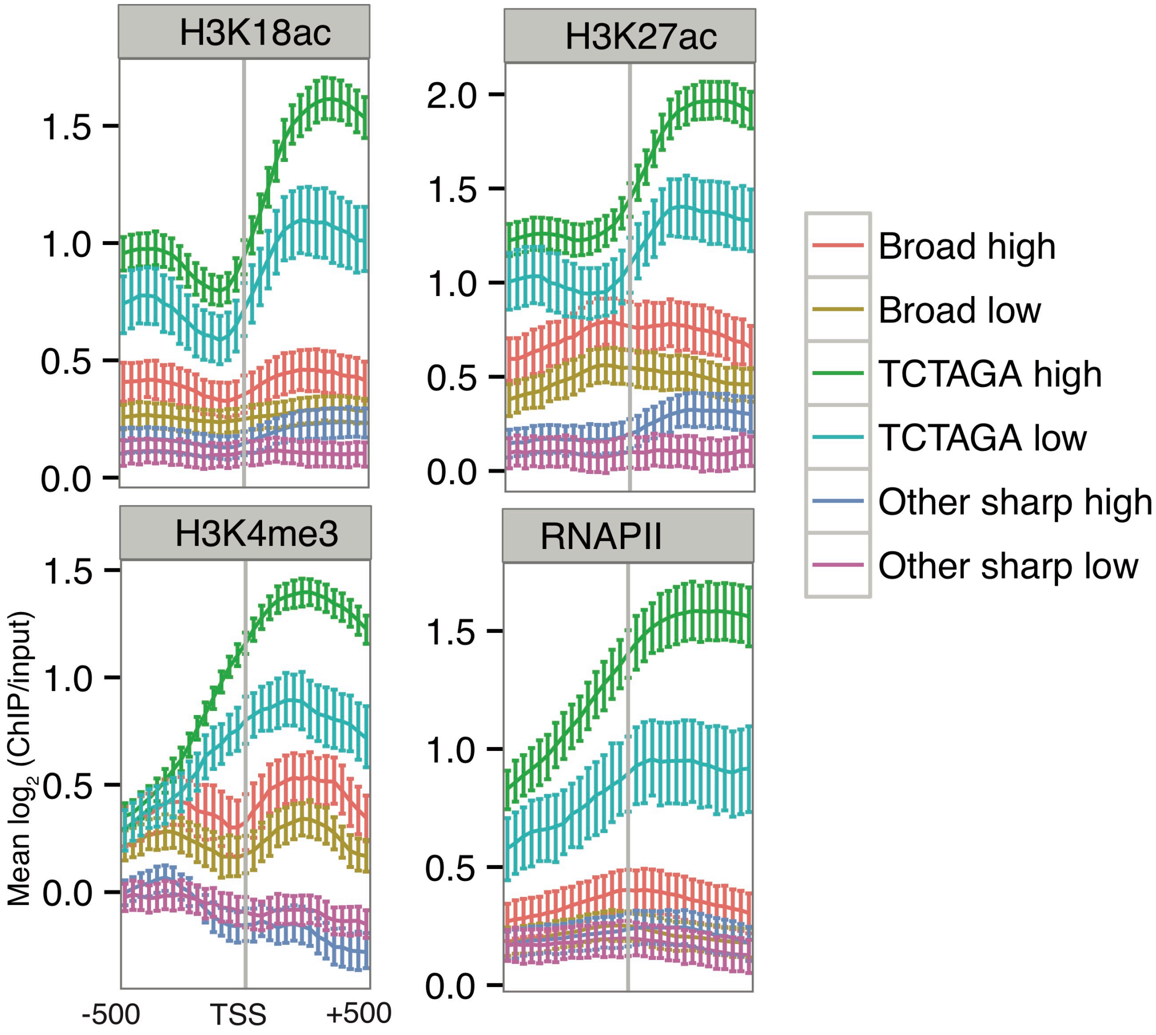

Figure S7.

Chromatin features of male-specific TCTAGA-promoters in the *O. dioica* testis. Data are shown for H3K18ac, H3K27ac, H3K4me3 and RNAPII ChIP-chip experiments. Each plot shows the mean $\log_2$ ratio of ChIP/input at each probe position in a 1000 bp window centered on the dominant CAGE TSS. Promoters were categorized according to promoter type (sharp=narrow region of TSSs; broad=dispersed region of TSSs) and activity (high indicates a tpm ≥ median; low indicates < median tpm). Sharp promoters were also subdivided according to the presence of a TCTAGA motif. Error bars show 95% confidence intervals for the mean obtained by bootstrapping. Regardless of expression level, TCTAGA-promoters have a higher enrichment for all features shown compared to promoters without this male-specific motif.

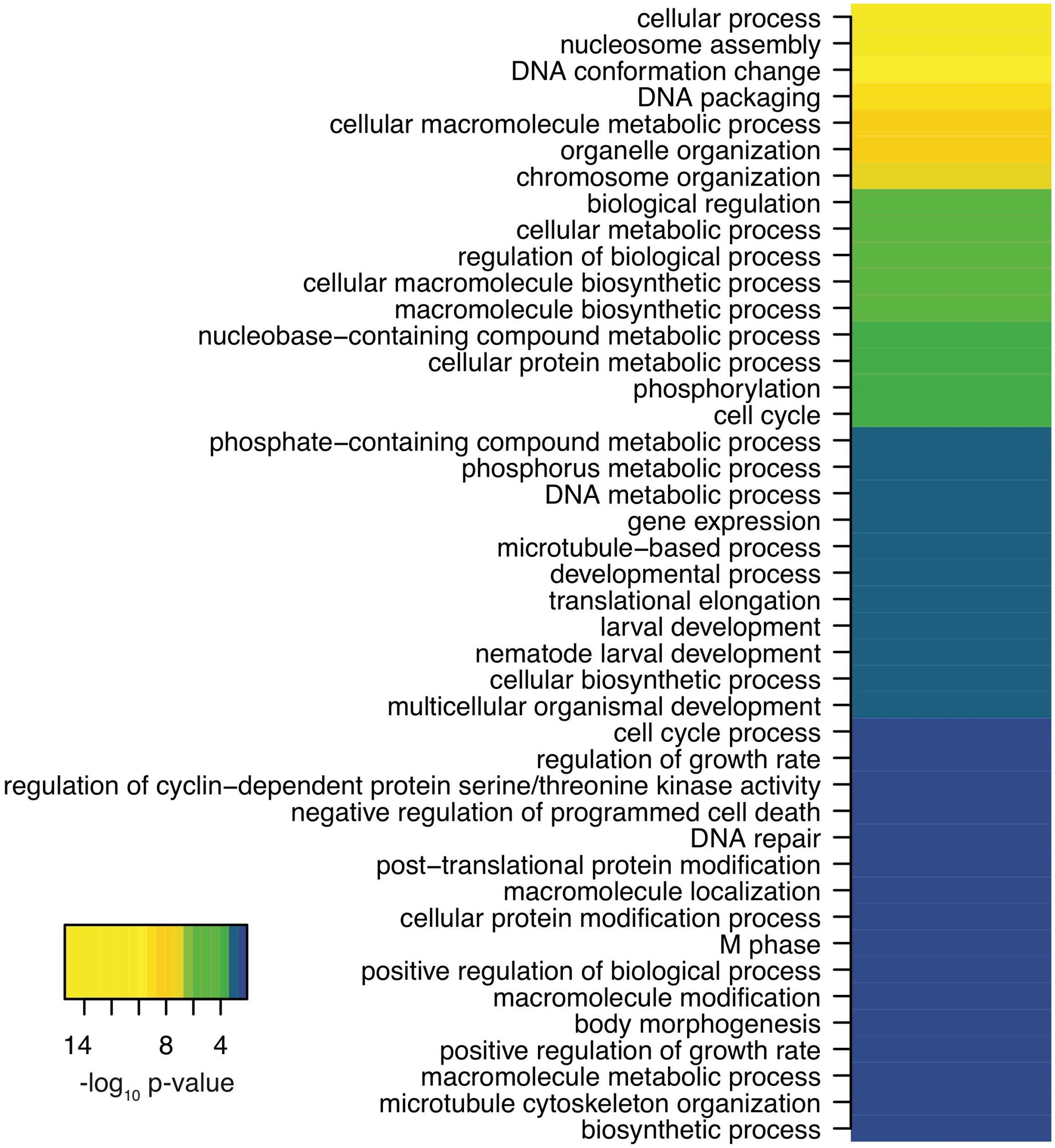

Figure S8.

Functions of E2F1-regulated genes. GO-terms enriched in genes associated with E2F1-bound broad promoters in the ovary, sorted and coloured according to respective *P*-values.

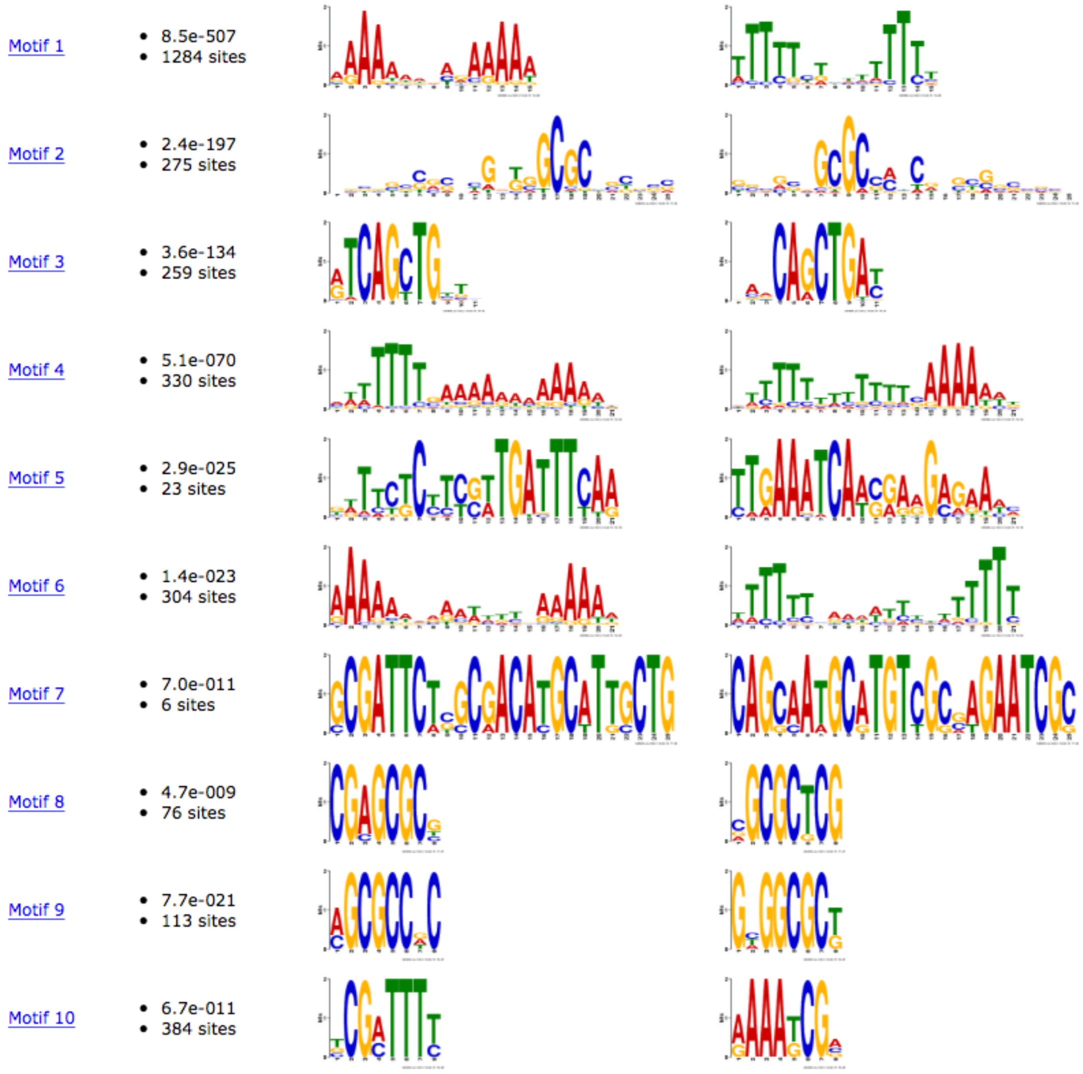

Figure S9.

Over-represented motifs found in zygotic promoters. Top 10 over-represented motifs discovered by MEME are shown for sequences in the 200 bp region surrounding zygotic TSSs (Tailbud TSSs in expression cluster 0_0). Motif 3 is a significant match to human TFAP4 (Figure 3C).

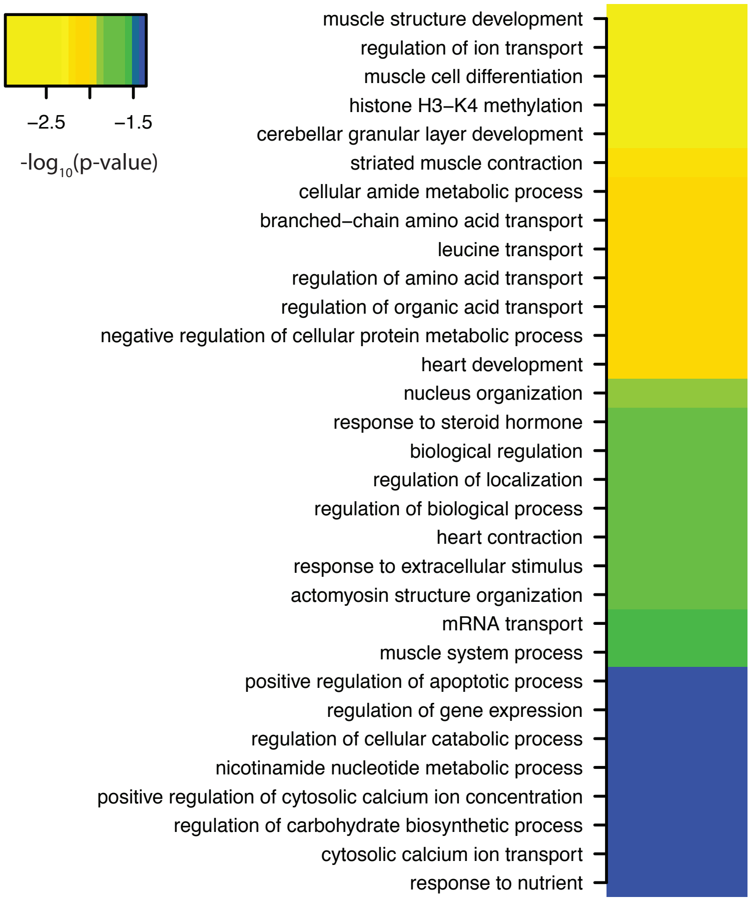

Figure S10.

Functions of genes with a TFAP4 promoter element. GO-terms enriched in zygotic genes with a TFAP4-like motif in their promoter regions in the tailbud, sorted and coloured according to respective *P*-values.

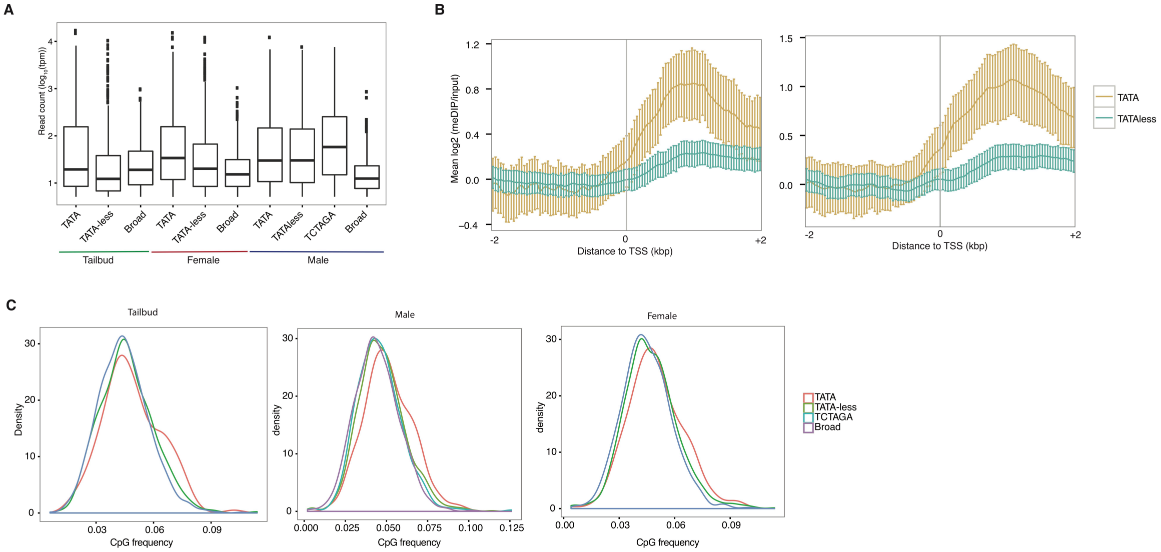

Figure S11.

DNA methylation, expression levels and downstream CpG content of TATA and TATA-less promoters. DNA-methylation enrichment downstream of TATA-dependent promoters is not explained by differing expression levels (**A**) and continues further downstream (**B**) but does coincide with a higher CpG content (**C**). Data in (**A)** and **(C**) correspond to the subset of promoters used in Figure 4. **(A)** Boxplot shows the distribution of expression levels ($\log_{10}$ tpm of dominant TSS) for broad promoters as well as sharp promoters with and without a TATA-element in the embryo (tailbud), male and female day 6 animals, and for promoters in the male with an upstream TCTAGA motif instead of a TATA-element. TATA-dependent and TATA-less promoters in the male did not have a significant difference in mean expression levels (Wilcoxon rank sum test: W = 142539.5, *P*-value = 0.9812). **(B)** Plots show the mean $\log_2$ ratio of methyl-DNA IP/input (y-axis) at each probe position (x-axis) in a 4 kb window centred on the dominant TSS, in two independent meDIP-chip biological replicates using *O. dioica* testes. Error bars show 95% confidence intervals for the mean obtained by bootstrapping. Each plot shows sharp promoters divided into those with a TATAA-element and those without. **(C)** Density plots show the distribution of CpG frequencies in the 500 bp region downstream of the dominant TSS for each subset of promoters in the embryo (tailbud), male and female animals.

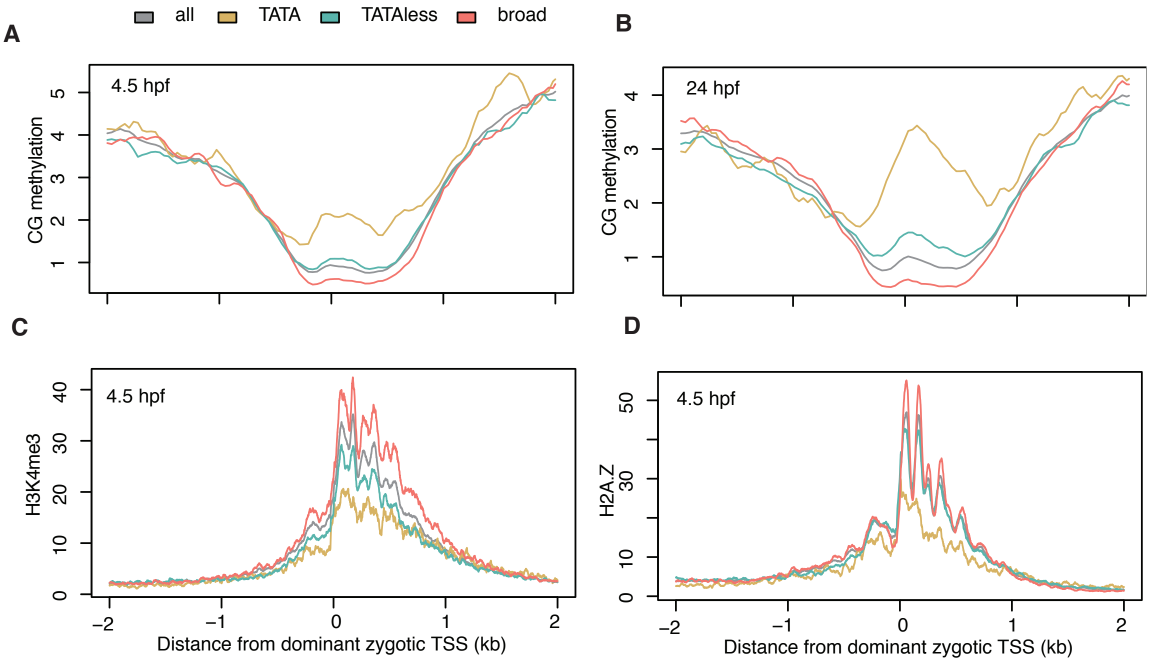

Figure S12.

TATA-dependent promoters have increased 5-methylcytosine DNA methylation at the TSS in zebrafish. Plots show the mean read count in a TSSs in a 4-kb window centered on zygotic dominant CTSSs using meDIP-seq data at 4.5 hpf (A) and 24 hpf (B), and H3K4me3 (C) and H2A.Z (D) ChIP-seq subtracted coverage data at dome/30% epiboly stage (4.33-4.66 hpf). In each plot the mean for all promoters, broad promoters and sharp promoters with and without a TATA-element are shown.

Supplemental References

1. Haberle V, Li N, Hadzhiev Y, Plessy C, Previti C, Nepal C, et al. Two independent transcription initiation codes overlap on vertebrate core promoters. Nature. 2014;507:381–5.

2. Nepal C, Hadzhiev Y, Previti C, Haberle V, Li N, Takahashi H, et al. Dynamic regulation of the transcription initiation landscape at single nucleotide resolution during vertebrate embryogenesis. Genome Research. 2013;23:1938–50.

3. Lee HJ, Lowdon RF, Maricque B, Zhang B, Stevens M, Li D, et al. Developmental enhancers revealed by extensive DNA methylome maps of zebrafish early embryos. Nat Comms. 2015;6:6315.

4. Zemach A, Mcdaniel IE, Silva P, Zilberman D. Genome-Wide Evolutionary Analysis of Eukaryotic DNA Methylation. Science. 2010;328:916–9.

5. Haberle V, Forrest ARR, Hayashizaki Y, Carninci P, Lenhard B. CAGEr: precise TSS data

retrieval and high-resolution promoterome mining for integrative analyses. Nucleic Acids Res. 2015;43:e51–1.

6. Danks GB, Raasholm M, Campsteijn C, Long AM, Manak JR, Lenhard B, et al. Trans-splicing and operons in metazoans: translational control in maternally regulated development and recovery from growth arrest. Molecular Biology and Evolution. 2015;32:585–99.

7. Yokomori R, Shimai K, Nishitsuji K, Suzuki Y, Kusakabe TG, Nakai K. Genome-wide identification and characterization of transcription start sites and promoters in the tunicate Ciona intestinalis. Genome Res. Cold Spring Harbor Lab; 2016;26:140–50.
